# Serum Cortisol as a Predictor of Major Adverse Pulmonary Event in Emergency Department Acutely Dyspneic Patients

**DOI:** 10.1155/2018/1758643

**Published:** 2018-10-11

**Authors:** Ozlem Dikme, Ozgur Dikme

**Affiliations:** ^1^Emergency Department, Koc University Hospital, Istanbul 34035, Turkey; ^2^Emergency Department, University of Health Sciences Istanbul Training and Research Hospital, Istanbul 34035, Turkey

## Abstract

Cortisol is a steroid hormone released from the adrenal glands in response to stressful conditions. Elevated cortisol levels have been described in stress, but it is unclear whether these are associated with adverse outcomes. In this study, we assess whether cortisol levels drawn in patients presenting with dyspnea to the ED were a predictor of major adverse pulmonary event (MAPE). In 87 patients presenting with dyspnea to the ED, cortisol levels were determined. Patients were then assessed to determine the following MAPE: endotracheal intubation (ETI) in the ED, admission to the intensive care unit (ICU), and in-hospital all-cause mortality. Forty-four patients (50.6%) were female and 33 (37.9%) were diagnosed with heart failure. Cortisol levels in patients with and without MAPE were 34.3*μ*g/dL and 23.8*μ*g/dL, respectively (p<0.001). Also, cortisol levels were found higher in patients intubated in the ED than nonintubated patients (54.2*μ*g/dL vs 25.7*μ*g/dL, p<0.001), higher in patients admitted to the ICU (38.7*μ*g/dL vs 24 *μ*g/dL, p<0.001), and higher in patients who died in hospital (50*μ*g/dL vs 24.3*μ*g/dL, p<0.001). The area under the ROC curve using cortisol to detect any component of MAPE—ETI or ICU admission or in-hospital all-cause mortality—was 0.76 (95% CI, 0.65-0.84). A cortisol value of 31.4*μ*g/dL had sensitivity of 70.8% and specificity of 79.4% for predicting MAPE. Patients in the MAPE group had higher serum cortisol levels than those without any MAPE. Cortisol may be used as a marker to predict MAPE in nontraumatic acutely dyspneic adult patients on ED admission.

## 1. Introduction

Dyspnea is a common symptom affecting up to the majority of adult patients admitted to emergency departments (EDs) and it may arise from a wide range of clinical conditions on the EDs such as heart failure, community-acquired pneumonia, chronic obstructive pulmonary disease, or asthma. According to the population-based studies, dyspnea prevalence has been found as 9 to 13% for adults and in the United States it accounts for 3 to 4 million ED visits annually. Diagnosis and treatment of the underlying cause of dyspnea is the preferred approach, but in the EDs there are many patients for whom the cause is unclear [1]. In this case, prediction of the outcome can be more significant than diagnosis. Specific biomarkers have diagnostic benefit in specific clinical disorders, but there is no prognostic biomarker for correlating nearly with changes in dyspnea across all cases [2].

Cortisol is a biochemical marker which is released from the adrenal gland with activation of the hypothalamic pituitary adrenal axis and plays a principal role in stressful conditions. In the literature already described some clinical conditions are found to be related to cortisol levels [3–10]. However, dyspnea can be considered one of the individual causes of the stress in the ED. Based on these hypotheses, in this study we investigated serum cortisol levels in patients admitted to the emergency department (ED) with acute nontraumatic dyspnea and its association with MAPE which included endotracheal intubation (ETI) in the ED, admission to the intensive care unit (ICU), and in-hospital all-cause mortality.

## 2. Material and Methods

### 2.1. Study Design

This prospective cross-sectional study was performed on nontraumatic acutely dyspneic patients who presented to the ED between the dates of September 1, 2013, and December 31, 2013, in a training and research hospital ED which has 180000 visits annually. Permission for the study was approved by the local ethics committee (305/02.08.2013).

Selection of participants: All consecutive patients over the age of 18 with nontraumatic acute dyspnea [dyspneic numeric rating scale (dNRS) ≥4] were assessed for the eligibility. The acutely dyspneic patient had to have shortness of breath either at rest, with exertion, or on lying down as a prominent complaint in the past week. Patients whose dyspnea was clearly a result of trauma (e.g., knife wounds, cardiac tamponade) were excluded. Patients with unstable angina or acute myocardial infarction (determined by ECG changes and cardiac enzymes) were excluded unless their predominant presentation was that of dyspnea. Additional exclusion criteria have been identified as using any form of steroid, prior hypo- or hypercortisolism (Cushing's syndrome/disease, pituitary tumor or ectopic tumor, Pseudo-Cushing's syndrome, Addison's disease, Nelson's syndrome, and Sheehan's syndrome), and pregnancy. When a decision was made for a patient to be included in the study, informed consent was obtained, and a blood sample was collected to measure the serum cortisol level.

### 2.2. Outcome Measures

The primary outcome variables were serum cortisol levels, which were used to predict MAPE (ETI in the ED, admission to the ICU, and in-hospital all-cause mortality).

### 2.3. Study Protocol

All patients were evaluated in the ED triage area and they took appropriate triage category regardless of the study settings. Age, gender, ED admission time, vital signs, and dNRS score were noted. Patients were examined by an emergency physician and preliminary diagnosis was reported. Patients were followed up with necessary further investigations as was clinically indicated to ascertain final diagnosis. MAPE was defined as ETI in the ED, admission to the ICU, and in-hospital all-cause mortality. They were used to measure primary goal substantially and compared with serum cortisol levels.

Blood samples were obtained using standard venipuncture techniques. Serum cortisol levels were measured with 0.5 ml volume blood samples collection in red top tube. If gross hemolysis or visible lipemia was seen, blood sample was repeated. The serum was stored at −80°C until assayed. Serum cortisol levels were measured with an immunochemistry analyzer (XP, Siemens Healthcare Diagnostics, Erlangen, Germany) using a solid-phase competitive method. All of the patients were followed up until being discharged from the hospital. Patient information was collected from medical records and by interviews.

### 2.4. Statistical Analysis

All calculations were analyzed with SPSS 15.0 for Windows statistical software (IBM, Armonk, NY). The Kolmogorov-Smirnov tests were used to analyze the normal distribution of the variables. Mann-Whitney U test and Kruskal-Wallis test were used to test for differences between and within groups. Differences in medians were computed with corresponding 95% confidence intervals (CIs). The p values for comparisons of categorical variables were generated by the chi-square test. The relationship between numerical variables did not provide a parametric test condition; Spearman correlation was performed. Also cutoff values calculated, and ROC curve analysis was performed. Area under the receiver operating characteristic curve (AUC) was used to quantify prognostic accuracy. All the statistical analysis was done with 95% CIs and p value <0.05 was considered statistically significant.

## 3. Results

**Figure 1:**
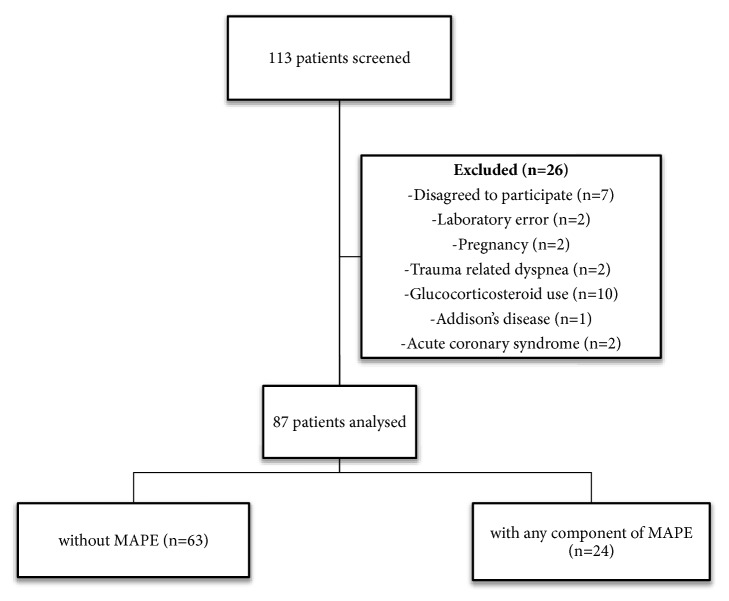
The patient flow diagram.

One hundred and thirteen consecutive patients were assessed for eligibility and 26 (23%) patients were excluded. Reasons of excluding patients are shown in Figure 1.

A total of 87 patients were included into the study. Of them, 44 (50.6%) were female and the mean age was 74 years (64-81). The leading diagnosis was heart failure (37.9%) followed by community-acquired pneumonia (33.3%) and chronic obstructive pulmonary disease or asthma (19.5%). Six patients (6.9%) were intubated in the ED and 19 patients (21.8%) were admitted to the ICU. Eight patients (9.2%), two in the ward and six in the ICU, died in hospital and five of them were also intubated in the ED. Median dNRS score was 8 (6-9) in patients with MAPE and was 6 (5-7) in patients without MAPE (p=0.001). Characteristics of study participants were shown in Table 1.

Median serum cortisol level in all patients was 26.7 *μ*g/dL (IQR: 14.9 to 34.1 *μ*g/dL). Serum cortisol level was 34.3 *μ*g/dL (22.5-50.2 *μ*g/dL) in patients with MAPE and 23.8 *μ*g/dL (13.7-29.7 *μ*g/dL) in patients without MAPE (p<0.001). Additionally, when the components of MAPE were analyzed separately, serum cortisol levels were found higher in patients intubated in the ED than nonintubated patients (54.2*μ*g/dL vs 25.7*μ*g/dL, p<0.001) and also higher in patients admitted to the ICU (38.7*μ*g/dL vs 24 *μ*g/dL, p<0.001) and in patients who died in hospital (50*μ*g/dL vs 24.3*μ*g/dL, p<0.001). The relationship between serum cortisol levels and MAPE was shown in Table 2.

The area under the ROC curve using cortisol to detect MAPE—ETI, or ICU admission, or in-hospital all-cause mortality—was 0.76 (95% CI, 0.65-0.84). A cortisol value of 31.4 *μ*g/dL had a sensitivity of 70.8% and a specificity of 79.4% for predicting MAPE. The area under the ROC curve using cortisol to detect ETI was 0.88 (95% CI 0.79-0.94), ICU admission was 0.78 (95% CI 0.68-0.86), and in-hospital all-cause mortality was 0.92 (95% CI 0.84-0.97). The ROC curve analyses for MAPE were also shown in Table 3.

## 4. Discussion

Dyspnea is defined as the sensation of a disability to breathe comfortably and it is a common chief complaint among ED patients. Evaluation of dyspneic patients is often difficult and diagnosis process is relatively long. Biomarkers are used widely to assist in diagnosis, risk stratification, treatment, and observation of recurrence in various disease processes, but there is no prognostic biomarker for correlating nearly with changes in dyspnea across all cases [2]. Prognostic utility of serum cortisol has also been known in critical illness. Pain, fever, hypovolemia, hypotension, and tissue damage all result in a constant increase in cortisol secretion and a loss of the normal diurnal variation [11]. However, dyspnea can be considered one of the individual causes of the stress in the ED. In the present hypothesis-generating study, we demonstrated that elevated serum cortisol level is associated with increased risk of MAPE in nontraumatic acutely dyspneic adult ED patients.

In the literature already described some clinical conditions are found to be related to cortisol levels. Cortisol excretion is proportional to the disease severity in hospitalized medical patients and this has already been reported in literature with positive correlation at some conditions [7] such as psychiatric disorders, viral infections, hypoglycemia, fever, surgery, and severe trauma [3–10]. Although some publications are also available, stating the opposite, studies reported that cortisol may be used as a predictor for cardiac risk event, adverse outcome, and mortality in specific clinical conditions [12–15]. Consistent with the literature, our study patients with MAPE have higher cortisol levels. Also, here we showed that patients with undifferentiated dyspnea without trauma who died in hospital or are intubated in the ED or admitted to ICU had higher serum cortisol levels upon initial presentation to the ED than patients who had none of MAPE.

## 5. Limitations

This study has several limitations. First, the sample size of our study was relatively small, and this was a single center study, which limits the generalization of our results. Second, we could not reach appropriately sample size of different diagnostic subgroups and we could not determine their cortisol levels changes (i.e., heart failure, community-acquired pneumonia). Finally, we measured only a single cortisol level in each acutely dyspneic patient, so we did not know any information about cortisol level peaks and treatment response.

## 6. Conclusions

Serum cortisol levels were higher in nontraumatic acutely dyspneic adult ED patients who were intubated in the ED or who were admitted to ICU or who died in hospital. Biomarkers can help determine the risk stratify and life-threatening conditions in the evaluation of the acutely dyspneic patient. Therefore, serum cortisol level may be used as a marker to predict MAPE in acute nontraumatic acutely dyspneic adult ED patients.

## Figures and Tables

**Table 1:** Characteristics of study participants.

	**All patients**	**MAPE**		**p**
	**(n=87)**	**Yes (n=24)**	**No (n=63)**	
Age, y, median (IQR)	74 (64-81)	74 (60-83)	74 (64-81)	0.837*∗*
Sex, Female, n (%)	44 (50.6%)	13 (54.2%)	31 (49.2)	0.679*∗∗*
Chronic disease, n (%)	77 (88.5%)	22 (91.7%)	55 (87.3)	0.720*∗∗*
dNRS, median (IQR)	6 (5-7)	8 (6-9)	6 (5-7)	0.001*∗∗∗*
Admission vital signs				
Hypotension, n (%)	6 (6.9%)	6 (25%)	0 (0)	<0.001*∗∗*
Hypertension, n (%)	34 (39.1%)	7 (29.2%)	27 (42.9%)	0.242*∗∗*
Tachycardia, n (%)	42 (48.3%)	18 (75%)	24 (38.1%)	0.002*∗∗*
Tachypnea, n (%)	26 (29.9%)	13 (54.2%)	13 (20.6%)	0.002*∗∗*
High temperature, n (%)	10 (11.5%)	0 (0)	10 (15.9%)	0.056*∗∗*
Low SpO2, n (%)	26 (29.9%)	12 (50%)	14 (22.2%)	0.011*∗∗*
MAPE				
ETI in the ED, n (%)	6 (6.9%)	6 (25%)	0 (0)	<0.001*∗∗*
ICU admissions, n (%)	19 (21.8%)	19 (79.2%)	0 (0)	<0.001*∗∗*
In-hospital mortality, n (%)	8 (9.2%)	8 (33.3%)	0 (0)	<0.001*∗∗*

Values are presented as median (IQR) or n (%). p values refer to Student t-test*∗*, chi square test*∗∗*, and Mann-Whitney-U test*∗∗∗*, as appropriate. Hypotension, systolic blood pressure <90 mm Hg; hypertension, blood pressure > 140/90 mm Hg; tachycardia, pulse rate > 100 beats/minute; tachypnea, respiratory rate > 20 breaths/minute; high temperature, >38 C; low SpO2, <90%. dNRS indicates dyspneic numeric rating scale.

**Table 2:** Relationship between cortisol levels and MAPE.

		**Cortisol** *μ*g/**dl**	**95**%** CI**	**p**
**MAPE**	**Yes (n=24)**	34.3	22.5-50.2	<0.001
	**No (n=63)**	23.8	13.7-29.7	

**ETI in the ED**	**Yes (n=6)**	54.2	42.2-83	<0.001
	**No (n=81)**	25.7	14.3-33.2	

**ICU admissions**	**Yes (n=19)**	38.7	23.7-50.9	<0.001
	**No (n=68)**	24	13.7-31.4	

**In-hospital mortality**	**Yes (n=8)**	50	37.8-64.9	<0.001
	**No (n=79)**	24.3	14.2-32.2	

Values are median (IQR); p values refer to Mann-Whitney U test. AUC, area under the receiver operating characteristic curve; CI, confidence interval; ETI, endotracheal intubation; ICU, intensive care unit.

**Table 3:** The ROC curve analyses for MAPE.

	**Cortisol, **	**Sensitivity, **%	**Specificity, **%	**+LR**	**-LR**	**AUC**
	***μ*** **g/dl**	**(95**%** CI)**	** (95**%** CI)**	**(95**%** CI)**	**(95**%** CI)**	**(95**%** CI)**
**MAPE**	31.4	70.8	79.4	3.43	0.37	0.76 (0.65-0.84)
		(48.9-87.3)	(67.3-88.5)	(2.6-4.6)	(0.2-0.8)	

**ETI in the ED**	46.3	83.3	92.6	11.25	0.18	0.88 (0.79-0.94)
		(36.1-97.2)	(84.6-97.2)	(7.8-16.2)	(0.03-1.3)	

**ICU admission**	31.4	73.7	76.5	3.13	0.34	0.78 (0.68-0.86)
		(48.8-90.8)	(64.6-85.9)	(2.3-4.2)	(0.1-0.8)	

**In-hospital all-cause mortality**	31.9	100	74.7	3.95	0	0.92 (0.84-0.97)
		(62.9-100)	(63.6-83.8)	(3.5-4.5)		

CI, confidence interval; ETI, endotracheal intubation; ICU, intensive care unit.

## Data Availability

No data were used to support this study.
